# 

**Published:** 1921-10

**Authors:** 


THE
### HOSPITAL and HEALTH REVIEW

Established 1885 No. 1843, Vol. LXXI.

New Series. Vol. I. No. 1 October 1921 Price Sixpence

SURGEON GENERAL'S LIBRARY
256998

CONTENTS.

| | PAGE |
|---|---|
| NOTES AND COMMENTS | 1 |
| PUBLIC HEALTH EDUCATION<br>Sir Arthur Newsholme, K.C.B., M.D. | 5 |
| THE TOXICITY OF PUTRID FOOD<br>W. G. Savage, M.D. | 11 |
| THE VOLUNTARY HOSPITALS<br>E. W. Morris, C.B.E. | 15 |
| REVIEW OF THE MONTH. JOHN KEATS, APOTHECARY AND POET | 17 |
| THE CHLORINATION OF WATER | 18 |
| PASSING EVENTS AND TOPICS | 19 |
| WOMEN IN NATIONAL LIFE<br>Mrs. E. M. Field. | 21 |
| THE MEDICAL ASPECTS OF DRIED MILK | 22 |
| LOCAL COMMITTEES | 24 |
| NOTABLE EPIDEMICS. I.—THE BLACK DEATH<br>A. T. Nankivell, M.D., D.P.H. | 25 |
| REVIEWS OF BOOKS | 27 |
| A LEAFLET COMPETITION | 29 |
| CORRESPONDENCE | 29 |

